# Post-fatigue fracture load, stress concentration and mechanical properties of feldspathic, leucite- and lithium disilicate-reinforced glass ceramics

**DOI:** 10.1016/j.heliyon.2023.e17787

**Published:** 2023-06-29

**Authors:** Vinicius Capobianco, Kusai Baroudi, Maria Jacinta Moraes Coelho Santos, José Henrique Rubo, Amin S. Rizkalla, Amanda Maria de Oliveira Dal Piva, Rafael Pino Vitti, João Paulo Mendes Tribst, Gildo Coelho Santos

**Affiliations:** a School of Dentistry, Herminio Ometto University Center, Araras, SP, Brazil; b RAK College of Dental Sciences, RAK Medical & Health Sciences University, RAS Al Khaimah, United Arab Emirates; c School of Dentistry, University of Taubaté, Taubaté, SP, Brazil; d Schulich School of Medicine & Dentistry, The University of Western Ontario, London, ON, Canada; e School of Dentistry, University of São Paulo, Bauru, SP, Brazil; f Department of Dental Materials, Academic Centre for Dentistry Amsterdam (ACTA),Universiteit van Amsterdam en Vrije Universiteit, Amsterdam, the Netherlands; g Department of Reconstructive Oral Care, Academic Centre for Dentistry Amsterdam (ACTA),Universiteit van Amsterdam en Vrije Universiteit, Amsterdam, the Netherlands

**Keywords:** CAD/CAM, Cementation, Dental materials, Mechanical properties., Finite element analysis

## Abstract

**Objective:**

To evaluate the mechanical properties of different CAD/CAM ceramic systems and the post-fatigue fracture and stress distribution when used as cemented crowns.

**Materials and methods:**

Sixty (60) CAD/CAM monolithic crowns were milled using three different ceramic materials (FD – Feldspathic [Vita Mark II]), LE - Leucite-based ceramic [IPS Empress CAD] and LD - Lithium Disilicate [IPS e.max CAD]) and adhesively cemented on resin composite dyes. Specimens were stored in distillated water (37 °C) for 7 days. After, half of the crowns were submitted to immediate fracture load test while the other half was submitted to fatigue cycling. The average cement layer of approximately 80 μm was assessed using scanning electron microscopy (SEM). The average thickness was used in the three-dimensional (3D) Finite Element Analysis (FEA). For each ceramic material, the density, Poisson ratio, shear modulus, Young modulus, fracture toughness, and true hardness were assessed (n = 3). The data was used to assess the Maximum Principal Stress throughout 3D-FEA according to each material during load to fail and post-fatigue. Data were submitted to two-way ANOVA and Tukey test (α = 0.05).

**Results:**

LD showed the highest compression load, density, shear modulus, Young modulus, fracture toughness and true hardness values. While LE presented the lowest mechanical properties values. There is no difference in the Poisson ratio between the evaluated ceramics.

**Conclusion:**

LD was susceptible to aging process but presented stronger physicomechanical properties, showing the highest post-fatigue fracture load and highest stress magnitude.

## 1 Introduction

Improving the aesthetic features of dental treatments has become an important goal for dentists and patients. Dental ceramics were first introduced in the 18th century and were originally used in dentures and individual teeth [1]. Thereafter, several dental ceramic systems have been developed and ceramic restorations became popular due to their optical properties, biocompatibility, wear resistance, color stability, and durability [1,2,3].

Ceramics are composed of metallic elements (aluminum, calcium, lithium, magnesium, potassium, sodium, lanthanum, tin, titanium and zirconium), non-metallic substances (silica, boron, fluorine, and oxygen) and must exhibit adequate properties such as natural tooth color, opacity, translucency, resistance to abrasion, and fracture toughness [1,2,4]. Dental ceramics can be processed using several techniques, such as stratification, heat pressing, 3D-printing and milling to fabricate crowns, inlays, onlays and ceramic veneers [1,4].

According to their microstructure, dental ceramics can be classified in glass-based/silica-based, glass-based with crystalline fillers, crystalline-based with glass fillers or polycrystalline solids [5]. Feldspathic ceramic is the glass-based ceramic most used for bi-layers restorations, and it is composed of feldspar (mix of potassium and sodium aluminosilicates), quartz (silica) and kaolinite (hydrated aluminosilicate) [2]. This type of ceramic is associated with better aesthetics [4] and was the first material (Vita Mark I, feldspathic ceramic block from Vita Zahnfabrik) used to produce CAD/CAM (Computer Aided Design/Computer Aided Manufacturing) restorations [5,6]. The advantage of that was that the crown could be produced in only one session (less chair time) [7], however lacking strength to be used in every case.

Later, CAD/CAM ceramic blocks have been available in different compositions, such as leucite crystals reinforced glass ceramics (KAlSi_2_O_6_) that are stronger than feldspathic but not failure-free [3,4,8]. To overcome this limitation, a lithium disilicate ceramic was developed for heat press technique and later for CAD/CAM, being suitable for crowns, inlays, onlays, veneers, three-unit bridges and implant-supported restorations [9]. Due to the advancement of materials processing and reliable CAD/CAM technology, feldspathic, leucite-reinforced glass ceramic and lithium-disilicate based ceramics are indicated to be used as monolithic crowns [10].

Monolithic ceramic crowns are commonly used due to their excellent esthetics and biocompatibility, but their mechanical behavior is different from that of natural teeth [10,11]. Ceramic materials are brittle and have low fracture toughness compared to natural teeth, which can lead to increased stress concentration and fracture susceptibility in certain areas of the crown [10,12]. Finite element analysis (FEA) is a computational technique that allows the simulation of the behavior of complex structures, such as dental crowns [10,13,14,15], under different loading and boundary conditions [10,12,14,16]. In addition, the presence of aging/fatigue testing on the evaluation of cemented ceramic crowns are crucial to ensure their long-term clinical success [10,17]. These tests can simulate the effect of long-term use and ensure that the crowns can withstand the stresses of daily use without failure. The results of these tests can also provide insights for improving the design and fabrication of cemented ceramic crowns.

Despite sharing the same clinical indication, different ceramic materials cannot always similarly behave in the same scenario [12]. The analysis of these materials after mechanical challenges provides valuable insight for controlling possible clinical failures, such as fracture. However, only with the assessment of mechanical properties can their behaviors be better evaluated. Therefore, the aim of this study was to evaluate the initial and post-fatigue fracture load of cemented CAD/CAM ceramic crowns, the physicomechanical properties of the restorative materials, as well as, their stress distribution. The null hypotheses would be that (i) the stress and physicomechanical properties have no difference between the evaluated CAD/CAM ceramics and (ii) the fatigue cycling has no effect on the fracture load of CAD/CAM crowns.

## 2 Materials and Methods

### 2.1 Crowns preparation

This study selected three different CAD/CAM ceramics: Feldspathic ceramic (FD - Vita Mark II, Bad Säckingen, Vita Zahnfabrik), Leucite-based ceramic (LC - IPS Empress CAD, Liechtenstein, Ivoclar), and Lithium Disilicate (LD - IPS e.max CAD, Liechtenstein, Ivoclar), totaling 60 crowns (n = 20). The shape of the crown was obtained from CEREC 3D (Operator's Manual, Sirona, 2001) program database and the cement thickness ranged between 50 and 150 μm. Therefore, a replica of a lower first molar to receive the ceramic crown followed the dimensions recommended by the system: 12 mm mesio-distal, 10 mm vestibular-lingual and 7 mm high. After milling (CEREC 2, Sirona for Dental Systems, Benshelm, Germany), all crowns were polished and cleaned. Next, LD crowns were crystalized in a ceramic oven according to the manufacturer's parameters.

**Fig. 1:**
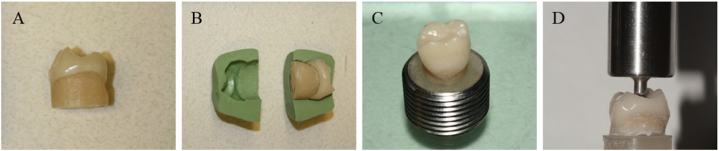
A) Composite abutment manufacturing according to the internal crown shape. B) Silicone matrix manufacturing for abutments standardization. C) The abutments were embedded in acrylic resin using a metal mold. D) Specimens under weight without the metal matrix.

The abutment teeth of mandibular first molars were manufactured with resin composite in order to obtain a substrate with mechanical properties similar to dentin (Tetric Evo Ceram, Liechtenstein, Ivoclar). For that, the resin was inserted into the inner part of one crown. Before the insertion of the photopolymerizable resin a relief was made in the inner part of the crowns with specific agents Tru-Fit silver color and gold color (Geo. Taub. Prod. & Fusion Co. Inc., Jersey City-NJ) and P.D.Q. Lubricant (Whip Mix, Canada) to create adequate space for subsequent insertion of the cement. After that, the increments were inserted and photopolymerized for 40 s each, until the reconstruction of the coronary part. After, a base of 1 cm in height was built. In sequence, a silicone matrix was made to maintain the standard format for all abutments. The abutments presented axial reduction of 2 mm, with functional and non-functional cusps reduced in 2 mm and 1.5 mm, respectively; facial and lingual walls with two plans, shoulder presenting 1 mm thickness and all angles were rounded. After, the specimens were embedded in an acrylic resin flat support, as summarized in Fig. 1 (A – D). Prior to cementation, each bonding surface of FD, LC and LD crowns was etched (hydrofluoric acid 5%), respectively during 60s, 60s and 20s. After, a layer of universal ceramic primer (Monobond Plus, Ivoclar) was applied. The abutments' bonding surface were treated with the mixture of Multilink Primer A + B (Ivoclar). Then, all crowns were adhesively cemented (Multilink Automix, Ivoclar), following the manufacturer's instructions. The resin cement was applied onto the intaglio surface of the crowns, which were then placed on the respective abutment and kept in a 750 g weight for 5 min. After removing the excess cement, the cement was light cured for 20 s on each face. All specimens were stored in distillated water at 37 °C for 7 days.

### 2.2 Immediate and post fatigue fracture load

Each group of crowns were subdivided into two subgroups (n = 10): immediate (control) and post-fatigue. The specimens of the immediate groups received a compression load (6 mm diameter, stainless steel) on occlusal fossa, transferring the load in tripoidism [10]. Each crown was loaded along its long axis with crosshead speed of 0.5 mm/min until complete bulk failure, using a universal testing machine (Instron model 3345, Norwood, MA, USA).

The other half of the crowns was subjected to a fatigue test using a universal testing machine (Instron model 8872, Norwood, MA, USA), where the specimens received a cycling axial load of 600 N [10] at 20Hz for 500.000 cycles [18] using the same load application as the immediate fracture load, in an aqueous environment. After, all crowns were transferred to the conventional universal testing machine (Instron model 3345) and were subjected to the compression test until failure, similar to the immediate group.

### 2.3 Scanning electron microscopy (SEM)

Two representative specimens were investigated under SEM analysis (LEO Zeiss 1540XB FEG-SEM, Zeiss, Oberkochen, Germany) to evaluate the cement layer. The specimens were coated with 7 nm of osmium metal and then mounted on SEM stubs with silver paint.

### 2.4 Mechanical properties assessment

From one block of each material, three (n = 3) slices (2 mm thickness, 1.4 mm height and 12 mm width) were cut vertically to calculate density, Poisson ratio, shear modulus, Young modulus, fracture toughness, and true hardness. For that, the ceramic slices were polished using 200- to 2000-grit sandpapers under water cooling.

#### 2.4.1 Young modulus, shear modulus and Poisson ratio

The Young modulus and Poisson ratio were calculated using the Ultrasonic method (V 665 oscilloscope, Hitachi, Japan), which used lithium niobate crystals for transmitting and receiving signals generated at 10 MHz of resonance frequency. Longitudinal and shear wave velocities were calculated from the thickness of the specimens and travelling time of the wave through the specimens [19]. The following (1), (2), (3), (4) were used to calculate the Young modulus:

(1) $$\frac{M1SG}{M1-M2}=$$

(2) $$E=\left[\frac{\left(1+v\right)\left(1-2v\right)}{1-v}\right]*\left(pC2L\right)$$

(3) G=pC2s

(4) $$v=\frac{\left[\frac{1}{2}*\left(\frac{CL}{Cs}\right)2-1\right]}{\left(\frac{CL}{Cs}\right)2-1}$$

Where *SG is* the specific gravity, *M*1 is the mass of specimen weighed in air, *M*2 is the mass of specimen weighed in the water, *ν* is the Poisson ratio, *p* is density of specimens (kg/m^3^), *C*L is the velocity of longitudinal wave across specimen (m/s), *C*s is the velocity of shear wave across specimen (m/s), *E* is the dynamic Young's modulus (GPa), and *G* is the dynamic shear modulus (GPa).

#### 2.4.2 Density

Density was calculated (n = 3) via the Archimedes method using an accurate balance (±0.1 mg). The density was calculated through the mass of the specimens in air divided per the difference between the mass of the specimens in air and in water, multiplied by the density of the water, which is temperature dependent.

#### 2.4.3 True hardness

Each specimen (n = 3) received a series of six Knoop indentations made by different loads ranging from 50g to 1000g using a microhardness unit (Buehler model 5114, Lake Bluff, IL, USA). The indentation length was plotted vs. the square root of the load values, and the true hardness was calculated from the slope of the linear regression line.

#### 2.4.4 Fracture toughness

To the fracture toughness evaluation (n = 3), 10 indentations were made on each specimen with a Vickers indenter using a microhardness unit (Buehler model 5114). On each specimen loads of 9.8 N (1000g) were applied for 15 s each. The indentation fracture toughness was calculated using formula (5) described below:

(5) $$K1c=H0\sqrt{\alpha }/\varphi [0.055\;\log*\left(\frac{8.4c}{\alpha }\right)*\left(\frac{H0}{E\varphi }\right)-0.4$$

Where, *KIc* is the indentation fracture toughness (MPa m^1/2^); *H0* is the true hardness (GPa), a half the diagonal of the Vickers indentation (mm); *E* is the dynamic Young modulus (GPa), and *φ* is the constraint factor.

### 2.5 Finite element analysis

**Fig. 2:**
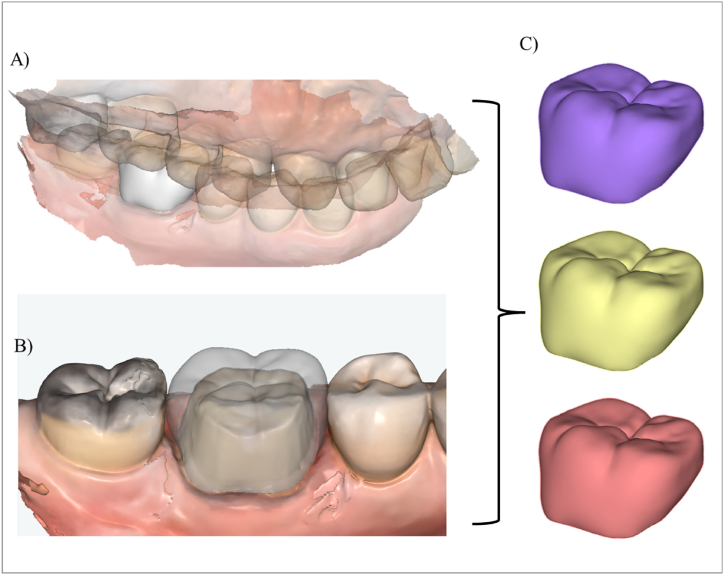
A and B) Molar crown designed in CAD software. C) Molar crowns geometries in Feldspathic ceramic (purple), Leucite-based ceramic (yellow), and Lithium Disilicate (red). (For interpretation of the references to color in this figure legend, the reader is referred to the Web version of this article.)

A numerical simulation using the 3D-finite element method was applied to calculate the stress concentration in different conditions, according to the crown restorative material and fatigue presence. The molar restoration file (Fig. 2A–C) designed at the Web-Based AI Dental CAD Software (Dentbird, Imagoworks Inc, South Korea, Seoul) was exported to the CAD software (Rhinoceros 5.0 – SR9 McNeil, North America) and edited to receive the cement and the preparation die, based on the crown internal shape. The cement layer was designed with 80 μm for all ceramics, based on the observed average with SEM. The volumetric solids were exported to the Computer-Aided Engineering software (ANSYS 19.2, ANSYS Inc., Houston, TX, USA) and replicated according to evaluated restorative materials (Fig. 2). The mesh convergence test was performed to define a mesh composed of 190,346 nodes and 88,260 tetrahedral elements.

**Fig. 3:**
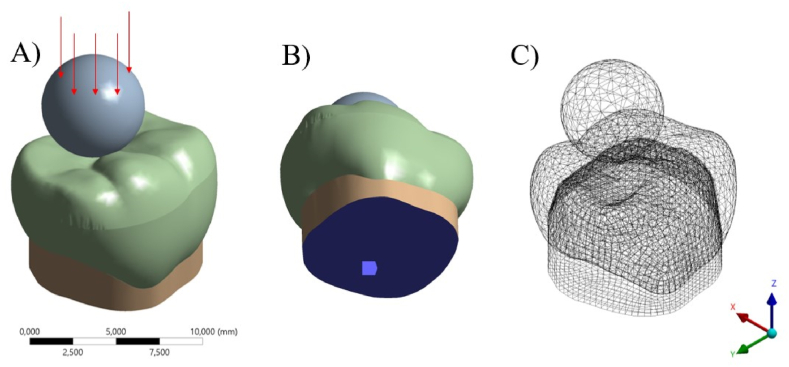
Finite element model with boundary conditions applied in the present simulation. A) Load application, B) fixation and C) mesh generation.

A static mechanical analysis was performed, in which all geometries were composed of homogeneous materials with linear and isotropic behavior. For the boundary conditions, the in vitro test average fracture load per group was used, totaling 6 models (3 materials with or without fatigue cycling) (Table 1). The set was fixed in the base of the abutment teeth with fixed zero nodal displacement, and the models were loaded with a 6 mm sphere (Fig. 3 A – C). Mechanical properties of the ceramic materials (Young modulus and Poisson ratio) were attributed according to the in vitro tests previously mentioned (Table 2); while resin cement and resin composite properties were respectively, 7 GPa; 0.45 [20] and 17 GPa; 0.28 [21]. Results were requested in Maximum Principal Stress (MPa) criteria for restoration. Maximum Principal Stress distribution was plotted in colorimetric stress maps and the stress peaks were obtained for quantitative comparison.

**Table 1:** Immediate and fatigued fracture load mean values ± standard deviation according to the ceramic material.

Ceramic	Presence of fatigue	Fracture load (N)	Stress peak (MPa)
Feldspatich ceramic - FD	No	1630.6 ± 508.6^B,a^	691.2
	Yes	1508.7 ± 520.2^B,a^	639.5
Leucite based ceramic - LE	No	1630.1 ± 629.3^B,a^	694.1
	Yes	1621.6 ± 425.0^B,a^	690.3
Lithium Disilicate - LD	No	2952.6 ± 545.0^A,a^	1256.5
	Yes	2154.4 ± 713.9^A,b^	916.8

Different small letters indicate statistically significant difference between immediate and fatigue fracture load. While capital letters indicate statistically significant difference between ceramics (ρ < 0.05).

**Table 2:** Mean values ± standard deviation of the evaluated mechanical properties according to the ceramic material.

Ceramic	Density (kg/m^3^)	Poisson ratio	Shear Modulus (GPa)	Young Modulus (GPa)	Fracture Toughness (MPa m^1/2^)	True Hardness (KH)
FD	2.45 ± 0.004^B^	0.15 ± 0.097^A^	28.12 ± 1.99^B^	64.57 ± 2.27^B^	2.42 ± 0.25^B^	4.09 ± 0.01^B^
LE	2.45 ± 0.006^B^	0.08 ± 0.031^A^	23.74 ± 0.75^C^	58.18 ± 1.67^C^	2.47 ± 0.05^B^	3.74 ± 0.09^C^
LD	2.48 ± 0.012^A^	0.22 ± 0.022^A^	44.19 ± 0.86^A^	96.14 ± 1.21^A^	3.79 ± 0.13^A^	4.87 ± 0.02^A^

Different letters indicate statistically significant difference between ceramics for each physicomechanical property (ρ < 0.05).

### 2.6 Statistical analysis

After assessing the data normality with the Kolmogorov-Smirnov test, the fracture load and mechanical properties data were submitted to two- and one-way ANOVA, respectively, and followed by Tukey's *post hoc* test at the significance level of α = 0.05.

## 3 Results

**Fig. 4:**
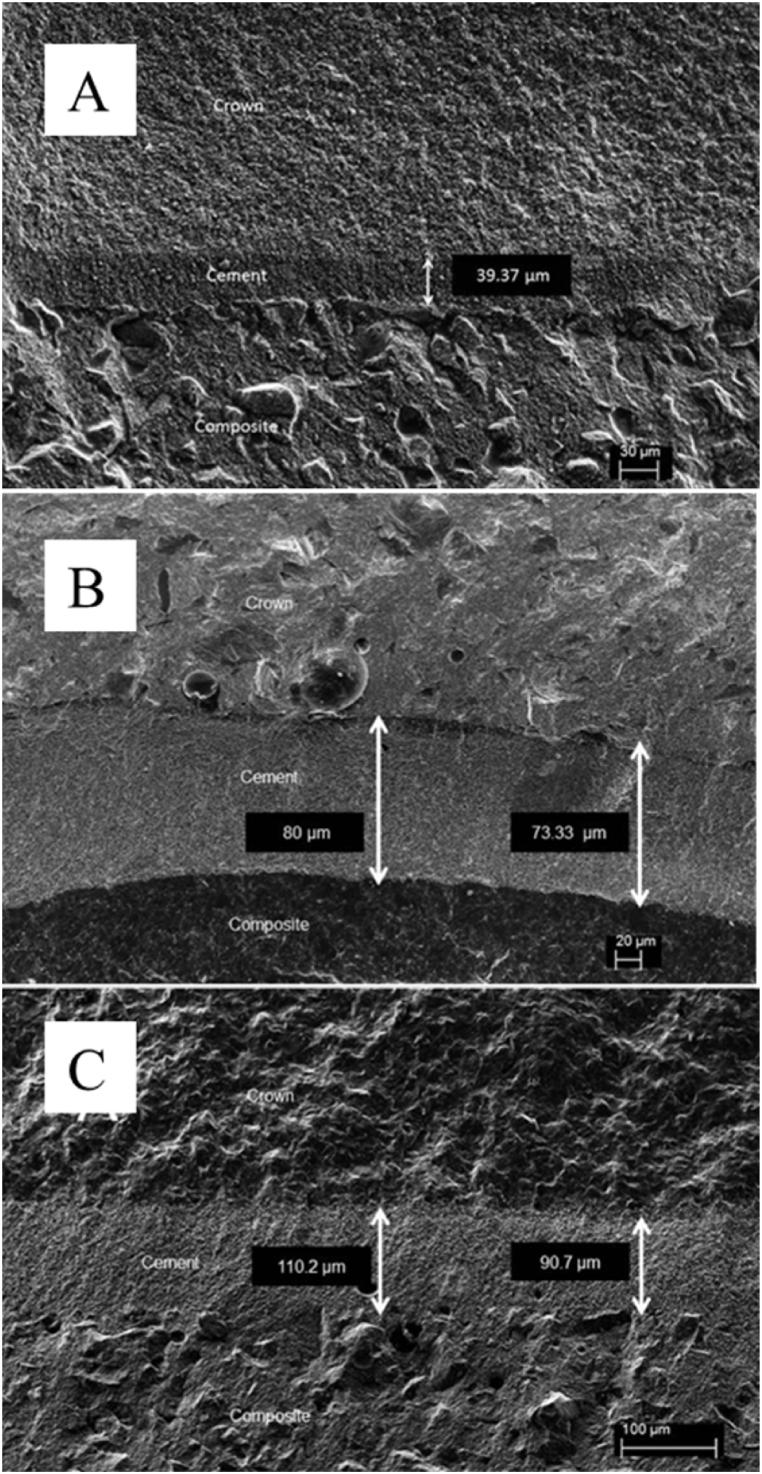
SEM analysis of A) Lithium Disilicate, B) Feldspathic ceramic, and C) Leucite-based ceramic.

Two-way ANOVA revealed that fatigue affected the fracture load only for LD (p = 0.03); while for the other evaluated ceramics, they showed similar immediate and fatigued compression load values (p = 0.62) (Table 1). Regarding crown material (p < 0.01), LD showed superior immediate fracture load, while it was similar to LE after fatigue. SEM micrographs of failed specimens show the cementation thickness of the LD, LC and FD, respectively. During SEM (Fig. 4), an average cement thickness values of representative specimens was evaluated to be considered in the FEA modelling, being approximately 80 μm (78 ± 6 μm).

Table 2 shows the physicomechanical properties of the investigated ceramics. LD showed superior mechanical properties (p = 0.000), except Poisson ratio (p = 0.08) which was similar for all materials. LE behaved like FD, except for Young modulus, shear modulus and true hardness (p = 0.01) in which LE showed inferior values.

**Fig. 5:**
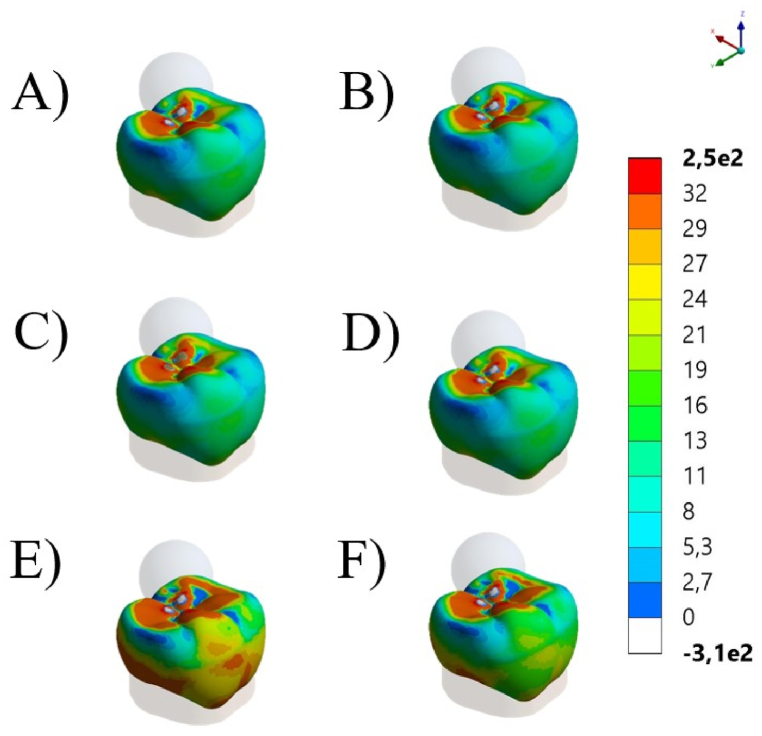
Isometric view of global contour plots of first principal stress (MPa) for each crown's material: Feldspathic ceramic before (A) and after aging (B), Leucite-based ceramic before (C) and after aging (D), and Lithium Disilicate before (E) and after aging (F).

**Fig. 6:**
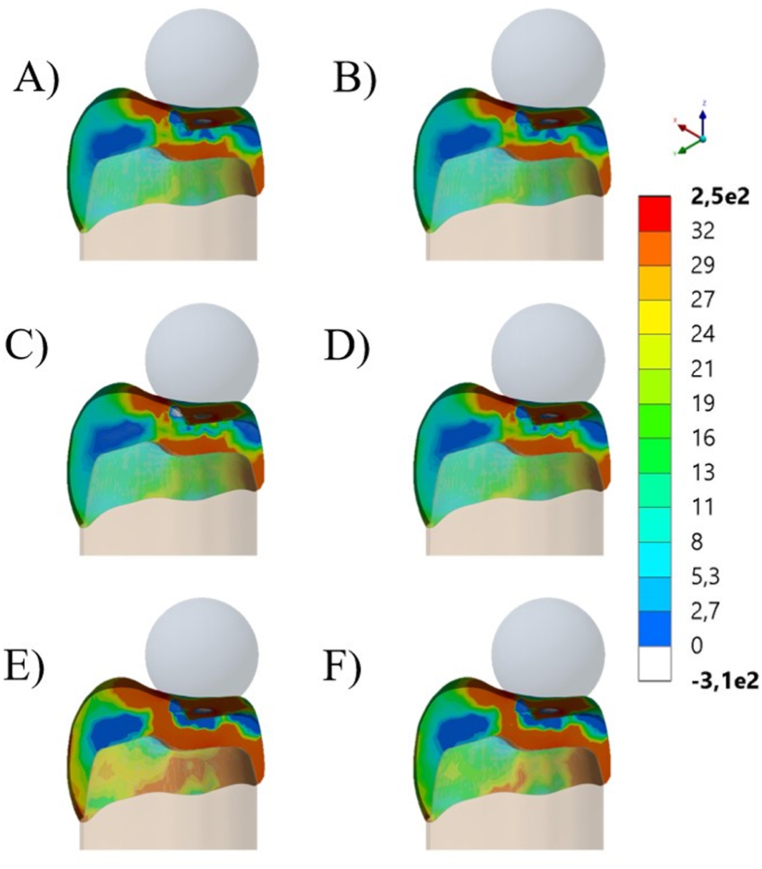
Section plane of global contour plots of first principal stress (MPa) for each crown's material. Feldspathic ceramic before (A) and after aging (B), Leucite-based ceramic before (C) and after aging (D), and Lithium Disilicate before (E) and after aging (F).

The stress maps for each simulated condition are summarized in Fig. 5, Fig. 6, respectively isometric and section plane views. The groups with FD and LE as restorative materials showed similar stress distribution patterns with higher stress concentration in two regions (cervical margin and intaglio's surface of the crown). For LD, stress concentration involves more areas of the crown, connecting cervical, intaglio and occlusal stresses. Additionally, since each model was loaded with the corresponding average fracture load, it is possible to observe that LD showed higher stress concentration during the fracture event, corresponding to its higher fracture strength. The stress peaks are summarized in Table 2, which were proportional to the observed fracture load.

## 4 Discussion

In the mouth, restorations could be loaded at several cycles which leads to a decreasing of its mechanical strength [22], which is in accordance with the results of this study (Table 1). The fatigue test had a significant negative effect on LD group when compared to not fatigated LD (Table 1). The remaining groups did not present significant differences among them, which could be partially explained by the cementation procedure and their mechanical properties. According to May et al. [23] crowns cemented with a thinner cement layer can withstand twice more load than crowns cemented with thicker layers. This can be observed in the representative SEM images, which presented a thinner cement layer thickness for LD compared to both other ceramics. According to the ceramic materials, LD presented the highest immediate and after aging fracture load. In addition, FD and LE presented more similar mean values for mechanical properties compared to the higher values of LD. Nevertheless, the adhesive/resin cementation had an important role on the fracture load of the ceramic restorations. The adhesive cementation leads to an increase on the mechanical strength and resistance of ceramics against fracture [24] and also balances the strength of less resistant ceramics, such as FD and LE restorations, compared to stronger ceramics, such as LD. This characteristic gives to the cementation procedure an important role on the dental treatments made in CAD/CAM ceramics. The thickness of the cement may influence the fracture strength of the evaluated ceramic crowns. Some different thicknesses of cement were observed (Fig. 4 A – C). However, this study only evaluated two representative specimens per group, not being strong enough to determine a layer profile for the groups. In addition, the differences in the measured thicknesses could be justified due to possible different evaluated areas. The influence of cement thickness on failure of CAD/CAM crowns was observed in a study using multi-physics finite element analysis and monotonic testing. Models were created to measure the load stress on a monolithic crown cemented with different resin cement thicknesses from 50 to 500 μm under occlusal load. It was observed that failure loads depended on cement thickness which reached: 673.5 N at 50 μm cement and 300.6 N at 500 μm [23]. It is worth to mention that despite the improvement of the restoration materials, the cementation technique is very important and must be carefully executed to achieve a thin layer of cement providing appropriate performance of the restorative material, in this case, CAD/CAM ceramic crowns.

Density is an important property of materials because it affects other properties, such as strength, hardness, elastic modulus and toughness. This is because higher density ceramics have fewer defects and imperfections. LD density can vary between 2.38 and 3.14 g/cm^3^ [25,26,27] while FD and LE show lower density values, respectively 2.4 ± 0.1 g/cm^3^ and 2.435 ± 0.001 g/cm^3^ [28,28]. Higher density ceramics tend to be harder due to a more tightly packed crystal structure, and tougher due to a more uniform microstructure that can better avoid crack propagation, which corroborates with higher toughness values for LD. However, the reported hardness and toughness values can be influenced by the testing conditions such as load, dwell time, and indenter geometry; which can make difficult the data comparison with previous studies [27,29,30]. In addition, higher density ceramics also tend to be stiffer because they have a higher elastic modulus, which is a measure of the material's resistance to deformation. The Young modulus of a dental material affects its ability to resist deformation and withstand stress, as well as its ability to distribute and absorb forces applied to it. In the present study, LD showed the most stiffer behavior (96.14 GPa), which is in the range of previously reported values (63.9 to 135.36 GPa) [26,27,30,31]. For FD, 58.18 GPa was also in the range from 48.7 to 70 GPa [26,27,30]; and LE (58.18 GPa) corroborated with previous reported value of 64.7 GPa [27]. Shear modulus, also known as the modulus of rigidity, describes the ratio of shear stress to shear strain in a material. It is a measure of a material's resistance to deformation under shear stress. A material with a high shear modulus will be more resistant to shear deformation and less susceptible to damage from shear stress. In this sense, LD would be the strongest one with 44.19 GPa. This result corroborate with previous studies that calculated 56.04 GPa for a ceramic material with partially similar structure [31]. Further studies evaluating these properties for the other dental ceramics should be developed to allow a better data comparison.

Poisson ratio is a measure of the relative deformation of a material in response to applied forces. It is defined as the negative ratio of the transverse strain (lateral contraction) to the longitudinal strain (axial extension) within the elastic limit. In the present study, the average value for LD was 0.22 corresponding with the exact value previously reported in the literature [26,30]. For LE, there are reports ranging from 0.17 [27] to 0.27 [32]. According to the present results of 0.08, this biomaterial is relatively incompressible or stiff in the transverse direction compared to the axial direction. The Poisson ratio for feldspathic ceramic was 0.23, however in other study it was measured at 0.15 [30]. The difference in values could have been caused by different methods of measurement. In summary, materials with a high Poisson ratio tend to contract laterally when stretched, while those with a low value tend to expand laterally when stretched. Regardless that, the assumption of Poisson's ratio equal to 0.3 is a common practice in finite element analysis, particularly in structural engineering and mechanics [6,10,12–15,23,26]. Therefore, it is advisable to use appropriate material properties based on experimental data or more accurate analytical models when available, rather than relying solely on general assumptions.

Table 2 shows the physicomechanical properties of the tested CAD/CAM ceramics. The advantage of these tests is that they require a small amount of material to perform the trials and produce sufficient data for analysis. It was noted that LD presented significantly higher physicomechanical properties when compared with LE and LD, respectively. These results are due to the composition of LD, which contains lithium disilicate crystals inside the glass matrix and also a different percentage of crystalline content, improving its fracture strength and acting as a toughening mechanism against the crack propagation [33], corroborating with the observed higher values of toughness, hardness and elastic modulus. Therefore, the first null hypothesis was rejected.

Fatigue of dental ceramics refers to the degradation or failure of dental ceramic restorations over time due to repeated loading and unloading cycles [10,18,20]. However, like all brittle materials, dental ceramics have a finite fatigue limit beyond which they will fail. Factors that can contribute to the fatigue of dental ceramics include the type and composition of the ceramic material, the design and preparation of the restoration, the fit and cementation of the restoration, and forces that the restoration is exposed to Refs. [10,20,32,33,34]. In addition, the presence of defects within ceramics can significantly decrease their fatigue survival, as these features act as stress concentration points that can initiate and propagate cracks [35]. Therefore, minimizing the presence of these features through careful manufacturing processes and material selection can improve the fatigue survival of ceramics. In this sense, fatigue cycling is a widely applied method in dental studies to simulate the long-term functional performance of dental restorations [34]. Subjecting the restorations to repeated cycles of loading and unloading, which simulates the biting and masticatory forces that they would be exposed to in the oral environment. In general, it is recommended that fatigue cycling tests should be designed to replicate the most severe and realistic clinical loading conditions as closely as possible. Data from clinical studies or patient surveys to estimate the number of cycles needed to simulate a certain period of time or number of masticatory cycles in the oral environment. However, it is important to note that there is no standardized or universally accepted protocol for estimating the number of cycles needed to perfectly simulate masticatory loads in an in vitro test [18,35].

According to the stress maps, all crowns present stress concentration in cervical margin, intaglio and occlusal surfaces; however, in different magnitudes. This is in accordance with previous FEA studies that have shown that the highest stresses are typically found in the cervical area of the crown [36,37], around the margin [12] and in the intaglio surface [10,38], which can lead to chipping, cracking, or debonding of the restoration [10,12]. In addition, the stress distribution in ceramic crowns is influenced by its material [10,12], design and thickness [4,6], and the direction and magnitude of the occlusal forces [14]. In this study, the average fracture load was applied to the correspondent group; therefore, LD showed larger red colored stress areas with higher stress peaks in comparison to similar behavior between FL and LE, which present closer elastic modulus values. In summary, regardless different physicomechanical properties and mechanical behavior, all tested ceramics can be considered suitable options for monolithic posterior crowns, since all evaluated crowns reached fracture load beyond above the physiological masticatory load in the posterior region (600 N) [39,40] and also parafunctional loads (>900 N) [41]. However, fatigue and stress corrosion could promote different outcomes.

Another study affirmed that the chewing forces can range from 9 to 180 N [42] while the maximum bite force for healthy young adults is within the range of 847 N for men and 597 N for women [43]. In patients with a very strong bite, both LE and LD would support approximately 2 times more load and LD approximately 3.5 times and 2.5 times before and after aging respectively. According to classical literature, the use of load-to-failure tests creates damage not seen clinically due to excessively high contact stresses [44,45]. In this sense, the present study showed with the stress analysis that with a proper loading application, stresses could be mainly concentrated at the intaglio's surface of the crown instead of only in the contacting region with the antagonist. Therefore, the present investigation applied the load-to-failure not as the unique outcome, but to investigate in agreement with FEA the stress caused in the moment of fracture. Merging the recorded load, mechanical properties and stress analysis, allowed to complement the literature and show the MPa for ceramic crowns when they are new and when they have been aged. This data would be the most approximate average of fracture strength (MPa) of a complex geometry represented by the molar.

As limitations, this study cannot make conclusions regarding cement layer thickness due to the limited number of specimens evaluated under SEM. In addition, it is important to emphasize that many clinical situations (*in vivo*) cannot be controlled and simulated in laboratory studies (*in vitro*) and they may affect final physicomechanical properties. Regarding the toughness results, the indentation technique has been discussed to be questionable due to sample and defects size effects, and lack of standardization; therefore, further investigations are also advocated to confirm or not the observed values. Finally, further studies evaluating other CAD/CAM ceramics, clinical conditions and cement layer thicknesses should be performed to investigate these variables, as well as, to evaluate ceramic restorations clinical longevity and fatigue life.

## 5 Conclusions

Based on the results of this study, it can be concluded that:

- Feldspathic and Leucite-based ceramics are weaker than Lithium Disilicate, however their fracture resistance remained stable after fatigue;
- Lithium Disilicate presented the strongest physicomechanical properties bearing more stress during the fracture event.

## 6 Clinical significance

Dentists and dental laboratory technicians should be careful to choose the right CAD/CAM ceramic for each clinical situation by taking into consideration their different physicomechanical properties.

## Author contribution statement

Vinicius Capobianco: Amin S. Rizkalla: Conceived and designed the experiments; Performed the experiments; Analyzed and interpreted the data; Wrote the paper.

Kusai Baroudi: Amanda Maria de Olveira Dal Piva: Analyzed and interpreted the data; Materials, analysis tools or data; Wrote the paper.

Maria Jacinta Moraes Coelho Santos: José Henrique Rubo: Conceived and designed the experiments; Contributed reagents, materials, analysis tools or data; Wrote the paper.

Rafael Pino Vitti: Conceived and designed the experiments; Analyzed and interpreted the data; Wrote the paper.

João Paulo Mendes Tribst: Conceived and designed the experiments; Performed the experiments; Analyzed and interpreted the data; Materials, analysis tools or data; Wrote the paper.

Gildo Coelho Santos Jr: Conceived and designed the experiments; Analyzed and interpreted the data; Contributed reagents, materials, analysis tools or data; Wrote the paper.

## Data availability statement

Data will be made available on request.

## Declaration of competing interest

The authors declare that they have no known competing financial interests or personal relationships that could have appeared to influence the work reported in this paper.

## References

[1] Kelly JR Benetti P. (2011). Ceramic materials in dentistry: historical evolution and current practice: ceramic materials in dentistry. Aust. Dent. J..

[2] Moshaverinia A. (2020). Review of the modern dental ceramic restorative materials for esthetic dentistry in the minimally invasive age. Dent. Clin..

[3] Peçanha M.M., Amaral M., Baroudi K., Frizzera F., Vitti R., Silva-Concilio L. (2022). Improving the bonding stability between resin cements and zirconia-based ceramic using different surface treatments. Int. J. Prosthodont. (IJP).

[4] Kasem A.T., Abo-Madina M., Tribst J.P.M., Al-Zordk W. (2023). Cantilever resin-bonded fixed dental prosthesis to substitute a single premolar: impact of retainer design and ceramic material after dynamic loading. J. Prosthodont. Res..

[5] Talibi M., Kaur K., Patanwala H.S., Parmar H. (2022). Do you know your ceramics? Part 1: classification. Br. Dent. J..

[6] Borges A.L.S., Tribst J.P.M., de Lima A.L., Dal Piva AM. de O., Özcan M. (2021). Effect of occlusal anatomy of CAD/CAM feldspathic posterior crowns in the stress concentration and fracture load. Clin. Exp. Dent. Res..

[7] Marchesi G., Camurri Piloni A., Nicolin V., Turco G., Di Lenarda R. (2021). Chairside CAD/CAM materials: current trends of clinical uses. Biology.

[8] Heintze S.D., Rousson V. (2010). Fracture rates of IPS Empress all-ceramic crowns--a systematic review. Int. J. Prosthodont. (IJP).

[9] Saavedra G., de S.F.A., Tribst J.P.M., Ramos N. de C., Melo RM de, Rodrigues V.A., Ramos G.F. (2021). Feldspathic and lithium disilicate onlays with a 2-year follow-up: split-mouth randomized clinical trial. Braz. Dent. J..

[10] Dal Piva AM. de O., Tribst J.P.M., Benalcázar Jalkh E.B., Anami L.C., Bonfante E.A., Bottino M.A. (2021). Minimal tooth preparation for posterior monolithic ceramic crowns: effect on the mechanical behavior, reliability and translucency. Dent. Mater..

[11] Pjetursson B.E., Sailer I., Latyshev A., Rabel K., Kohal R.-J., Karasan D. (2021). A systematic review and meta-analysis evaluating the survival, the failure, and the complication rates of veneered and monolithic all-ceramic implant-supported single crowns. Clin. Oral Implants Res..

[12] Dal Piva AM. de O., Tribst J.P.M., Borges A.L.S., Souza R.O., de A.E., Bottino M.A. (2018). CAD-FEA modeling and analysis of different full crown monolithic restorations. Dent. Mater..

[13] Celik H.K., Koc S., Kustarci A., Rennie A.E.W. (2022). A literature review on the linear elastic material properties assigned in finite element analyses in dental research. Mater. Today Commun..

[14] Ausiello P., Di Lauro A.E., Tribst J.P.M., Watts D.C. (2023). Stress distribution in resin-based CAD-CAM implant-supported crowns. Dent. Mater..

[15] Tribst J.P.M., Dal Piva A.M.O., Madruga C.F.L., Valera M.C., Borges A.L.S., Bresciani E., de Melo R.M. (2018). Endocrown restorations: influence of dental remnant and restorative material on stress distribution. Dent. Mater..

[16] Houdaifa R., Alzoubi H., Jamous I. (2022). Three-dimensional finite element analysis of worn molars with prosthetic crowns and onlays made of various materials. Cureus.

[17] Tribst J.P., Dal Piva A.O., Madruga C.F., Valera M.C., Bresciani E., Bottino M.A., de Melo R.M. (2019). The impact of restorative material and ceramic thickness on CAD\CAM endocrowns. J. Clin. Exp. Dent..

[18] Fraga S., Rodrigues C.D.S., Zucuni C.P., Pereira G.K.R., Barbosa M.N., Valandro L.F., May L.G. (2020). High load frequency at 20Hz: its effects on the fatigue behavior of a leucite-reinforced glass-ceramic. J. Mech. Behav. Biomed. Mater..

[19] Rizkalla A.S., Jones D.W. (2004). Indentation fracture toughness and dynamic elastic moduli for commercial feldspathic dental porcelain materials. Dent. Mater..

[20] Demachkia A.M., Sichi L.G., Rodrigues J.V., Nogueira Junior L., Araújo R.M., Ramos N.C., Bottino M.A., Tribst J.P.M. (2022). Implant-supported restoration with straight and angled hybrid abutments: digital image correlation and 3D-Finite Element Analysis.". Eur. J. Gen. Dent..

[21] Puškar T., Jevremović D., Blažić L., Vasiljević D., Pantelić D., Murić B., Trifković B. (2010). Holographic interferometry as method for measuring strain caused by polymerisation shrinkage of dental composite. Contemp. Mater..

[22] Valandro L.F., Cadore-Rodrigues A.C., Dapieve K.S., Machry R.V., Pereira G.K.R. (2023). A brief review on fatigue test of ceramic and some related matters in Dentistry. J. Mech. Behav. Biomed. Mater..

[23] May L.G., Kelly J.R., Bottino M.A., Hill T. (2012). Effects of cement thickness and bonding on the failure loads of CAD/CAM ceramic crowns: multi-physics FEA modeling and monotonic testing. Dent. Mater..

[24] Hallmann L., Ulmer P., Kern M. (2018). Effect of microstructure on the mechanical properties of lithium disilicate glass-ceramics. J. Mech. Behav. Biomed. Mater..

[25] Salman S.M., Salama S.N., Mahdy E.A. (2019). Crystallization characteristics and properties of lithium germanosilicate glass-ceramics doped with some rare earth oxides. Bol. Soc. Esp. Ceram. Vidr..

[26] Trindade F.Z., Valandro L.F., de Jager N., Bottino M.A., Kleverlaan C.J. (2018). Elastic properties of lithium disilicate versus feldspathic inlays: effect on the bonding by 3D Finite Element Analysis. J. Prosthodont..

[27] Belli R., Wendler M., de Ligny D., Cicconi M.R., Petschelt A., Peterlik H., Lohbauer U. (2017). Chairside CAD/CAM materials. Part 1: measurement of elastic constants and microstructural characterization. Dent. Mater..

[28] Palacios T., Tarancón S., Pastor J.Y. (2022). On the mechanical properties of hybrid dental materials for CAD/CAM restorations. Polymers.

[29] Yan J., Kaizer M.R., Zhang Y. (2018). Load-bearing capacity of lithium disilicate and ultra-translucent zirconias. J. Mech. Behav. Biomed. Mater..

[30] Ramos Nde C., Campos T.M., Paz I.S., Machado J.P., Bottino M.A., Cesar P.F., Melo R.M. (2016). Microstructure characterization and SCG of newly engineered dental ceramics. Dent. Mater..

[31] Biskri Z.E., Rached H., Bouchear M., Rached D. (2014). Computational study of structural, elastic and electronic properties of lithium disilicate (Li_2_Si_2_O_5_) glass-ceramic. J. Mech. Behav. Biomed. Mater..

[32] Della Bona A., Mecholsky J.J., Anusavice K.J. (2004). Fracture behavior of lithia disilicate-and leucite-based ceramics. Dent. Mater..

[33] Schultheis S., Strub J.R., Gerds T.A., Guess P.C. (2013). Monolithic and bi-layer CAD/CAM lithium-disilicate versus metal-ceramic fixed dental prostheses: comparison of fracture loads and failure modes after fatigue. Clin. Oral Invest..

[34] da Rosa L.S., Velho H.C., Tribst J.P.M., Valandro L.F., Kleverlaan C.J., Pereira G.K.R. (2023). Weak adhesion between ceramic and resin cement impairs the load-bearing capacity under fatigue of lithium disilicate glass-ceramic crowns. J. Mech. Behav. Biomed. Mater..

[35] Zhang Y., Sailer I., Lawn B.R. (2013). Fatigue of dental ceramics. J. Dent..

[36] Zheng Z., Sun J., Jiang L., Wu Y., He J., Ruan W. (2022). Influence of margin design and restorative material on the stress distribution of endocrowns: a 3D finite element analysis. BMC Oral Health.

[37] Sichi L.G.B., Pierre F.Z., Arcila L.V.C., de Andrade G.S., Tribst J.P.M., Ausiello P. (2021). Effect of biologically oriented preparation technique on the stress concentration of endodontically treated upper central incisor restored with Zirconia crown: 3D-FEA. Molecules.

[38] Kharboutly N.A.-D., Allaf M., Kanout S. (2023). Three-dimensional finite element study of endodontically treated maxillary central incisors restored using different post and crown materials. Cureus.

[39] D'souza K.M., Aras M.A. (2017). Three-dimensional finite element analysis of the stress distribution pattern in a mandibular first molar tooth restored with five different restorative materials. J. Indian Prosthodont. Soc..

[40] Ha S.R. (2015). Biomechanical three-dimensional finite element analysis of monolithic zirconia crown with different cement type. J. Adv. Prosthodont..

[41] Teixeira F.M., de Assis Claro C.A., Neves A.C., de Mello Rode S., da Silva-Concílio L.R. (2012). Influence of loading and use of occlusal splint in implant-supported fixed prostheses. J. Craniofac. Surg..

[42] De Long R., Douglas W.H. (1983). Development of an artificial oral environment for the testing of dental restoratives: bi-axial force and movement control. J. Dent. Res..

[43] Waltimo A., Könönen M. (1993). A novel bite force recorder and maximal isometric bite force values for healthy young adults. Scand. J. Dent. Res..

[44] Kelly J.R., Benetti P., Rungruanganunt P., Bona A.D. (2012).

[45] Kelly J.R. (1999). Clinically relevant approach to failure testing of all-ceramic restorations. J. Prosthet. Dent.

